# Cardiothoracic ratio measurement using artificial intelligence: observer and method validation studies

**DOI:** 10.1186/s12880-021-00625-0

**Published:** 2021-06-07

**Authors:** Pairash Saiviroonporn, Kanchanaporn Rodbangyang, Trongtum Tongdee, Warasinee Chaisangmongkon, Pakorn Yodprom, Thanogchai Siriapisith, Suwimon Wonglaksanapimon, Phakphoom Thiravit

**Affiliations:** 1 Department of Radiology, Faculty of Medicine Siriraj Hospital, Mahidol University, 2 Wanglang Road, Bangkoknoi, Bangkok, 10700 Thailand; 2 Institute of Field Robotics, King Mongkut’s University of Technology Thonburi, Bangkok, Thailand

**Keywords:** Cardiothoracic ratio, Deep learning, Clinical validation, Observer variation, AI

## Abstract

**Background:**

Artificial Intelligence (AI) is a promising tool for cardiothoracic ratio (CTR) measurement that has been technically validated but not clinically evaluated on a large dataset. We observed and validated AI and manual methods for CTR measurement using a large dataset and investigated the clinical utility of the AI method.

**Methods:**

Five thousand normal chest x-rays and 2,517 images with cardiomegaly and CTR values, were analyzed using manual, AI-assisted, and AI-only methods. AI-only methods obtained CTR values from a VGG-16 U-Net model. An in-house software was used to aid the manual and AI-assisted measurements and to record operating time. Intra and inter-observer experiments were performed on manual and AI-assisted methods and the averages were used in a method variation study. AI outcomes were graded in the AI-assisted method as excellent (accepted by both users independently), good (required adjustment), and poor (failed outcome). Bland–Altman plot with coefficient of variation (CV), and coefficient of determination (R-squared) were used to evaluate agreement and correlation between measurements. Finally, the performance of a cardiomegaly classification test was evaluated using a CTR cutoff at the standard (0.5), optimum, and maximum sensitivity.

**Results:**

Manual CTR measurements on cardiomegaly data were comparable to previous radiologist reports (CV of 2.13% *vs* 2.04%). The observer and method variations from the AI-only method were about three times higher than from the manual method (CV of 5.78% *vs* 2.13%). AI assistance resulted in 40% excellent, 56% good, and 4% poor grading. AI assistance significantly improved agreement on inter-observer measurement compared to manual methods (CV; bias: 1.72%; − 0.61% *vs* 2.13%; − 1.62%) and was faster to perform (2.2 ± 2.4 secs *vs* 10.6 ± 1.5 secs). The R-squared and classification-test were not reliable indicators to verify that the AI-only method could replace manual operation.

**Conclusions:**

AI alone is not yet suitable to replace manual operations due to its high variation, but it is useful to assist the radiologist because it can reduce observer variation and operation time. Agreement of measurement should be used to compare AI and manual methods, rather than R-square or classification performance tests.

## Introduction

Chest radiography (CXR) is the most widely-used modality for screening of lung and heart diseases in clinical practice due to its easy accessibility and cost-effectiveness [1]. The cardiothoracic Ratio (CTR) obtained from CXR is the preferred index to provide prognostic information on heart disease [1–4]. The CTR is derived from a ratio of heart to internal thoracic diameters with a value of more than 0.5 indicating the presence of cardiomegaly [2]. Manual calculation of CTR introduces observer variation and is time consuming. Therefore, automatic calculation of CTR could be a useful tool for clinicians and radiologists to improve accuracy and reduce workload.

Deep Learning (DL), a subset of Artificial Intelligence (AI) methods, has demonstrated advances in medical imaging [5–8]. DL techniques can reliably/accurately classify CXR abnormalities [8], and detect diabetic retinopathy in fundus images [6]. The technique has also been employed to automatically calculate CTR [9–12]. Presently, all DL techniques in CTR calculation are based on the U-Net model, the most successful convolutional network for biomedical image segmentation [13]. Since its conception, U-Net has inspired many successors and previous studies have reported that modification of encoding architecture can further improve accuracy [14–17]. While DL techniques in CTR calculation have been technically validated, only two reports [9, 11] with small sample size (n = 100) were conducted in the clinical setting. Therefore, there is a need to clinically validate this calculation technique with a large dataset before it can be implemented in routine hospital settings.

We aimed to assess CTR measurement agreement using manual and AI methods on both observer and method variations in a large dataset, and to investigate the clinical utility of the AI method. Specifically, we assessed if the AI method could be used independently and how much time it could save compared to the manual approach. We also investigated agreement of measurement, linear correlation, and classification performance tests as indicators to determine if the AI method could replace manual operations.

## Materials and methods

### Study population

This study complied with the Declaration of Helsinki and was approved by the Siriraj Institutional Review Board (Si069/2020). Informed consent was waived due to the retrospective nature of the study. There were two data groups, normal and cardiomegaly, in the study. Data were acquired from chest x-ray radiologist reports between 2010–2019 from patients aged over 17 years, and their PA-upright CXR images were retrieved from the Picture Archiving Communication System (PACS) in our radiology department. Normal chest x-rays, and chest x-rays with cardiomegaly with CTR measurements were included. Five-thousand normal CXR images were randomly obtained and all 2,517 cardiomegaly images were acquired for a total of 7,517 images. The CTR values from radiologist reports were considered to be the reference method. Reference CTR values were only available for the cardiomegaly data because in our high patient volume clinical setting, radiologists measure CTR only on cases suspected of having cardiomegaly.

### AI model

**Fig. 1:**
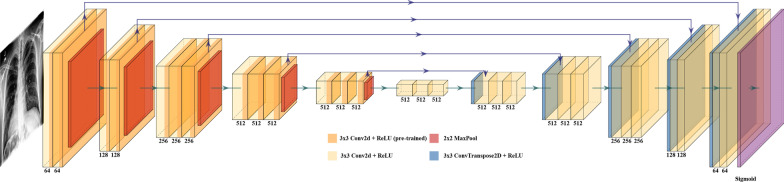
Model architecture of U-Net with VGG-16, the AI model used in this experiment

The deep learning technique employed in this study was based on U-Net with VGG-16 encoding. The model was an in-house development project and a collaboration between radiologists and machine learning scientists [10]. Due to the lack of high-quality open-source solutions and the shortage of large, open datasets with high-quality segmentation annotations, we adopted a well-known U-Net model architecture and then trained the model using public datasets. U-Net leverages feature pooling to generate context and successive up-sampling operators to achieve a high-resolution mask output (Fig. 1). The network consists of two connecting parts; the down-sampling part also referred to as the encoder or the contraction path, and the up-sampling part also known as the decoder or the expansion path. The contraction path uses successive convolutional layers with max pooling that consecutively reduces the input dimensions. This encoding part is replaced with VGG-16. The expansion path utilizes a stack of up-sampling blocks, each block containing an up-sampling operation followed by a 2 × 2 up-convolution. The output of each block is concatenated with the feature maps from the corresponding layer of the encoder, and parsed through two consecutive convolutions for output assembly.

**Fig. 2:**
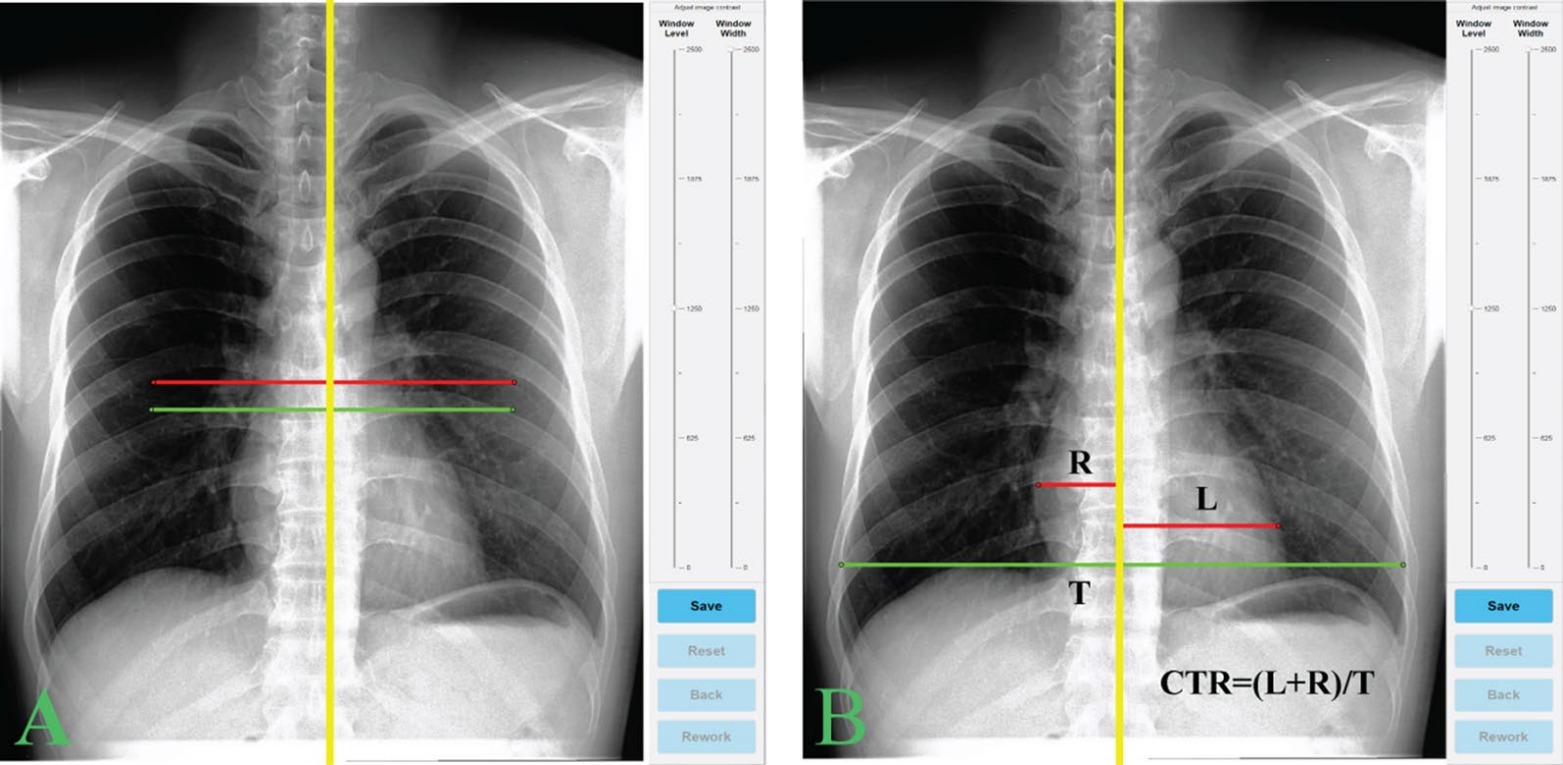
An in-house software for CTR measurement. The green and red lines are heart and chest border lines at default (**a**) and user-adjusted (**b**) positions, and the spine line is yellow. CTR was calculated from the ratio of these heart and chest lines (**b**)

The network was trained using a limited public dataset [10], and manually labeled by radiologists and non-experts with 2,002 samples with ground-truth heart segmentations and 1,238 samples with ground-truth lung samples. In the CTR calculation process, the network produces thoracic and cardiac masks. The left and right extreme points of these masks were used to find heart and lung diameters and to calculate the heart and lung ratio or CTR (Fig. 2b). Our data were not part of a training or validation process for the DL model, and so functioned as a test of the model itself [18]. Finally, the technique was considered to have given a failed outcome when it could not segment the lung or heart (i.e., the technique gave unreasonable lung or heart size, such as heart size less than 3 mm).

### Experimental setting

The study was designed to investigate observer and method variations of CTR measurements between manual and AI techniques, which were performed on both normal and cardiomegaly data groups (all data) and only on cardiomegaly group to emulate normal clinical practice at our hospital. Three CTR measurement methods were applied; manual, AI-assisted and AI-only. AI-only methods obtained CTR values from the U-Net with VGG-16 encoding model. A medical scientist with experience in medical image processing (PY) and a second-year radiology resident (KR) independently performed CTR measurements in the manual and AI-assisted methods with supervision from four experienced chest radiologists (TT, TS, WS, and PT). The independent measurements were performed separately and two weeks apart on each dataset to reduce measurement bias. PY performed the measurement twice (intra-observer), KR performed the measurement once, and the average of these three measurements was used for method variation studies.

We developed a program using MATLAB software (R2019a, MathWorks, Inc., Natick, MA, USA) to assist the user operations as shown in Fig. 2a. The software provides graphical user interface for CTR measurement and records the user-interaction time of each measurement. In the manual method, users were presented with three lines of heart and chest borders in a default position (Fig. 2a) and asked to adjust these lines to the appropriate locations (Fig. 2b). In the AI-assisted method, the lines were positioned as suggested by the AI calculation and users could choose to accept them without further adjustment or to disagree, which required adjustment of the lines. If there were any failure in the AI calculations, then the default line positions from the manual method were used. Based on human/user interaction in the AI-assisted method, AI outcomes could be categorized into excellent, good, and poor categories. Any AI calculation failure was classified as poor and its data were excluded from the variation and correlation experiments. When AI outcomes were accepted by both users independently, it was classified as excellent. Finally, if any adjustment was required by a user then the outcome was considered to be good. The time of each case was measured from the start of line adjustment to acceptance (*i.e.*, hit the save button as in Fig. 2b).

### Statistical analysis

Statistical analysis was performed on MATLAB and MedCalc (19.5.3, MedCalc software Ltd, Ostend, Belgium) software. The paired Student’s t-test was used for parametric evaluation between measurement methods with the statistical significance level set at p < 0.05. Bland–Altman plot and linear correlation were employed to evaluate agreement and correlation between measurement methods, respectively. Coefficient of variation (CV) signifying level of agreement was calculated from the standard deviation of the differences between two methods, then divided by their mean and expressed as a percentage. Thus, the lower the CV the better agreement was between two measurement methods. Coefficient of determination (R-Squared or R^2^) was defined into four categories: poor (less than 0.5), moderate (0.5 – 0.75), good (0.75–0.9), and excellent (more than 0.9). Finally, the performance of the cardiomegaly classification test was evaluated using accuracy, sensitivity, specificity, area under receiver operating characteristics curve (AUC), and F1-score metrics on CTR cutoff values at 0.5 (the standard), the optimum (*i.e.*, maximize both sensitivity and specificity), and the maximum sensitivity (*i.e.*, to rule out cardiomegaly).

## Results

### Patient characteristics

There were 4,933 (1,431 males and 3,502 females; aged 42.5 ± 14.8 years) patients with normal CXRs, and 2,419 (675 males and 1,744 females; aged 64.2 ± 14.0 years) CXRs from patients with cardiomegaly (Table 1). According to the radiologist reports (reference method), the mean CTR value in the cardiomegaly group was 0.569 ± 0.047.

**Table 1:** Patient demographic data

	Normal group	Cardiomegaly group
Number of patients	4,933	2,419
*Gender*		
Male	1,431 (29%)	6,75 (28%)
Female	3,502 (71%)	1,744 (72%)
Mean age (years)	42.5 ± 14.8	64.2 ± 14.0
*Age*		
< 18	33 (0.7%)	0 (0%)
18–35	1,771 (35.9%)	87 (3.6%)
36–50	1,562 (31.7%)	300 (12.4%)
51–65	1,225 (24.8%)	831 (34.3%)
66–80	329 (6.7%)	926 (38.3%)
> 80	13 (0.2%)	275 (11.4%)
CTR value	0.454 ± 0.043	0.569 ± 0.047

### AI outcomes

**Fig. 3:**
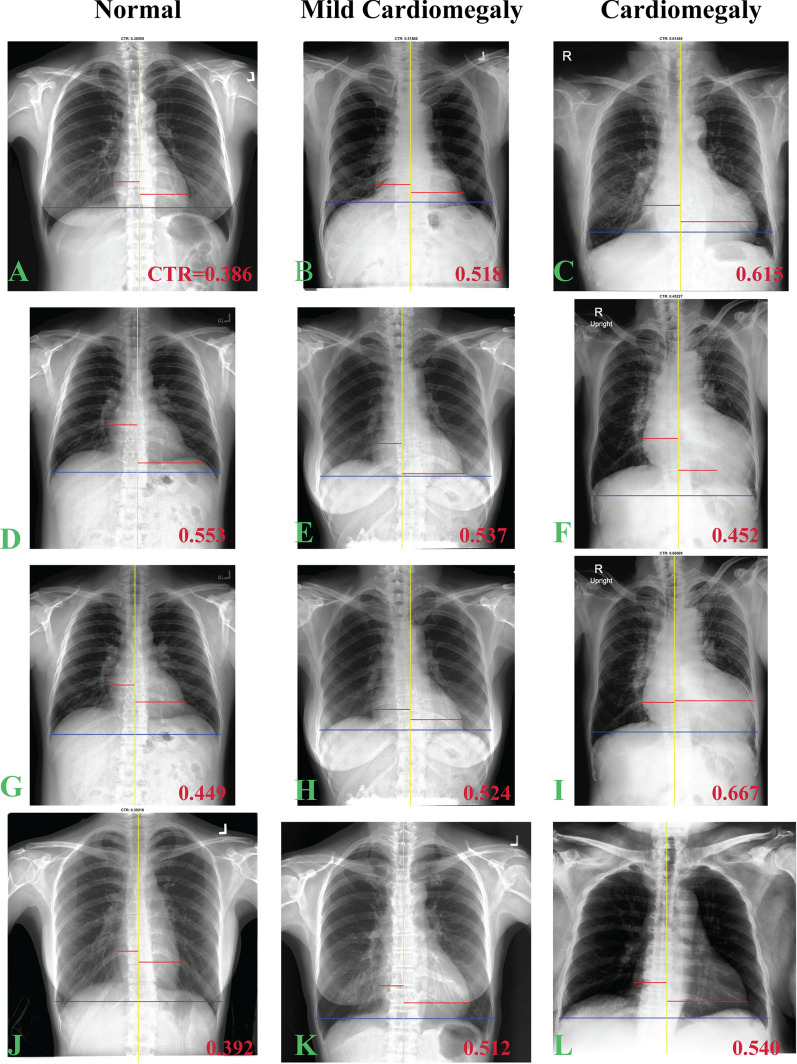
CTR measurements on normal, mild cardiomegaly and cardiomegaly cases using AI-only (the first and second rows) and manual (the third and fourth rows) methods. The first (**a**–**c**) and second (**d**–**f**) rows are CTR measurements by AI-only which accepted and rejected by user, respectively. The third row (**g**–**i**) is the user adjustment of the rejected AI-only operation (the second row) while the last row (**j**–**l**) demonstrates the failed cases from AI-only operation required fully manual operation. CTR value is displayed at the lower right corner of each image

With the AI-assisted method, 40% of outcomes were excellent, 56% were good, and 4% were poor (Fig. 3). Poor outcomes were mostly (97%; 290/299 cases) observed in the normal group, while only nine of 2,571 cardiomegaly cases had a poor outcome. Furthermore, most failures involved the heart segmentation calculation. Three examples of the poor outcome are displayed in Fig. 3j–l, which also illustrates CTR measurements from the AI-assisted method.

### Observer variations

**Fig. 4:**
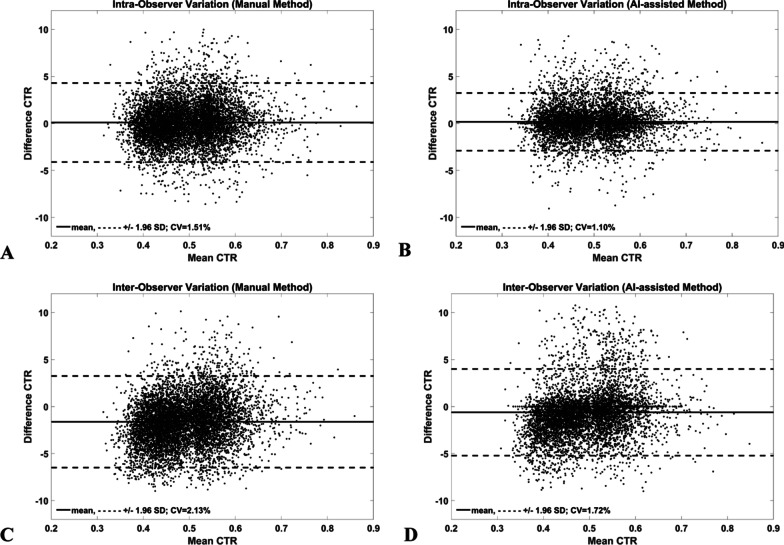
Bland–Altman plots of Manual (**a**, **c**) and AI-assisted (**b**, **d**) methods. Note: AI-assisted method had lower bias and CV as compared to Manual method on both intra- and inter-observer variation studies

Intra- and inter-observer variations from the manual and AI-assisted methods are presented in Fig. 4 and Table 2. Overall, the CV and bias of observer variations from both methods was lower than 2.2% and 1.7%, respectively, while the inter-observer variation of the manual method was 2.13%(CV) and − 1.62% (bias). The AI-assisted method significantly (p < 0.001) improved agreement on inter-observer measurements compared to the manual method (CV; bias: 1.72%; − 0.61% *vs* 2.13%; − 1.62%). Observer variations in the normal and the cardiomegaly group were comparable to that of both groups combined (data not shown). Therefore, the AI-assisted method increased observer agreement compared to the manual method.

**Table 2:** Bias, 95% CI, and coefficient of variation of intra- and inter-observer CTR measurements from Manual and AI-assisted methods on normal and cardiomegaly dataset

Method	Intra-observer		Inter-observer	
	Bias (95% CI) (%)	CV(%)	Bias (95% CI) (%)	CV (%)
Manual	0.09 (−4.10 4.29)	1.51	−1.62 (−6.50 3.25)	2.13
AI-assisted	0.17 (−2.90 3.23)	1.10	−0.61 (−5.22 3.99)	1.72

### Method variations

CTR values from manual, AI-only, and AI-assisted methods were not significantly different on normal (0.455 ± 0.043, 0.447 ± 0.058, and 0.453 ± 0.044, respectively) and cardiomegaly (0.570 ± 0.045, 0.569 ± 0.049, and 0.570 ± 0.044, respectively) data. They also did not differ from the reference method (0.569 ± 0.047). Variations (CVs) from the reference method to manual and AI-assisted methods were at a similar level to inter-observer variation of the manual method (CVs of 2.04% and 2.23% *vs* 2.13, respectively). Our CTR measurements on cardiomegaly data, hence, were comparable to the previous reports by experienced radiologists.

**Fig. 5:**
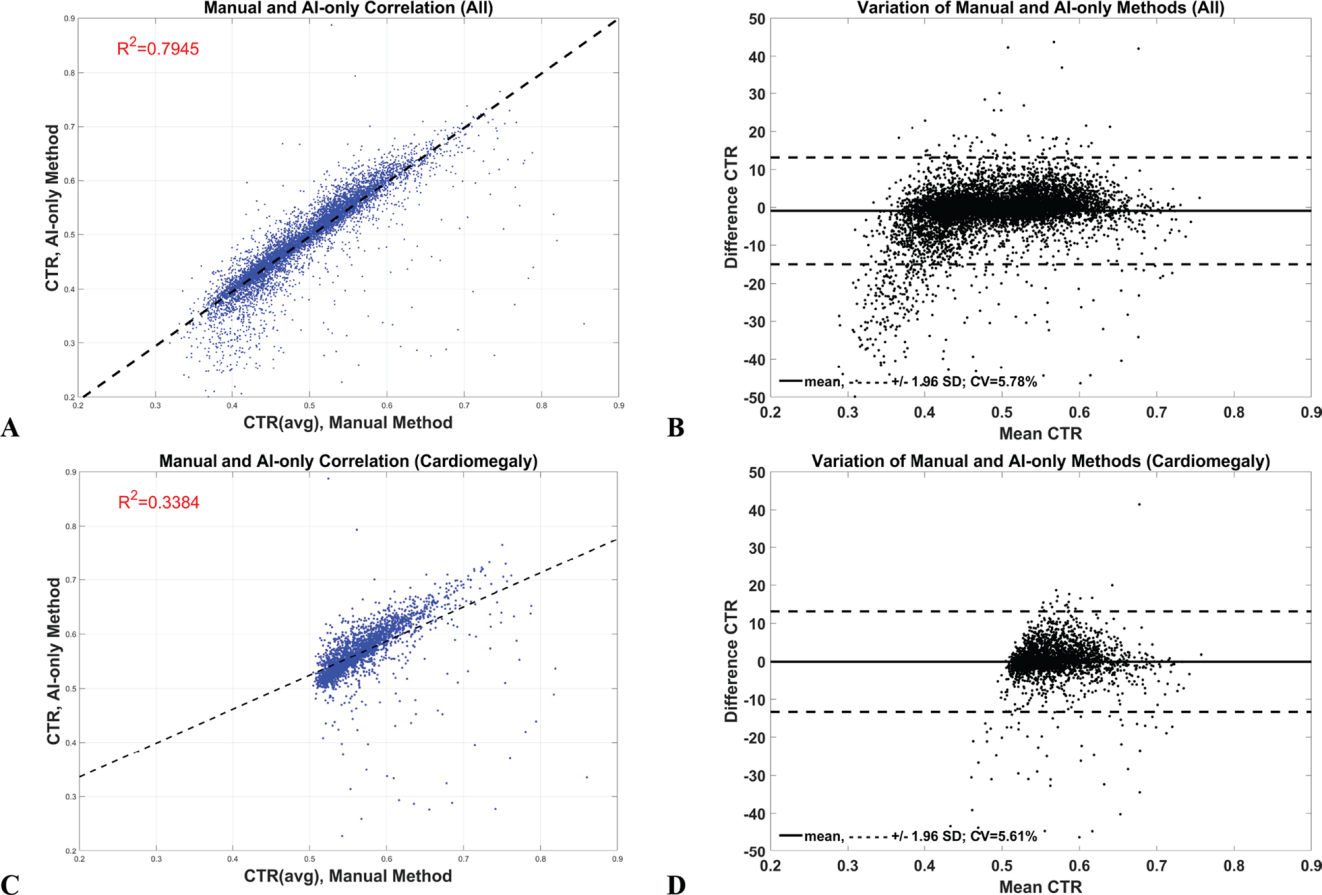
Linear correlation (**a**, **c**) and Bland–Altman (**b**, **d**) plots of Manual and AI-only method on all (the first row) and cardiomegaly (the last row) data. Note: Even these comparisons had high variation on both types of data, their R-squared were interestingly different as in good (0.7945) and poor (0.3384) in all and cardiomegaly data, respectively

**Fig. 6:**
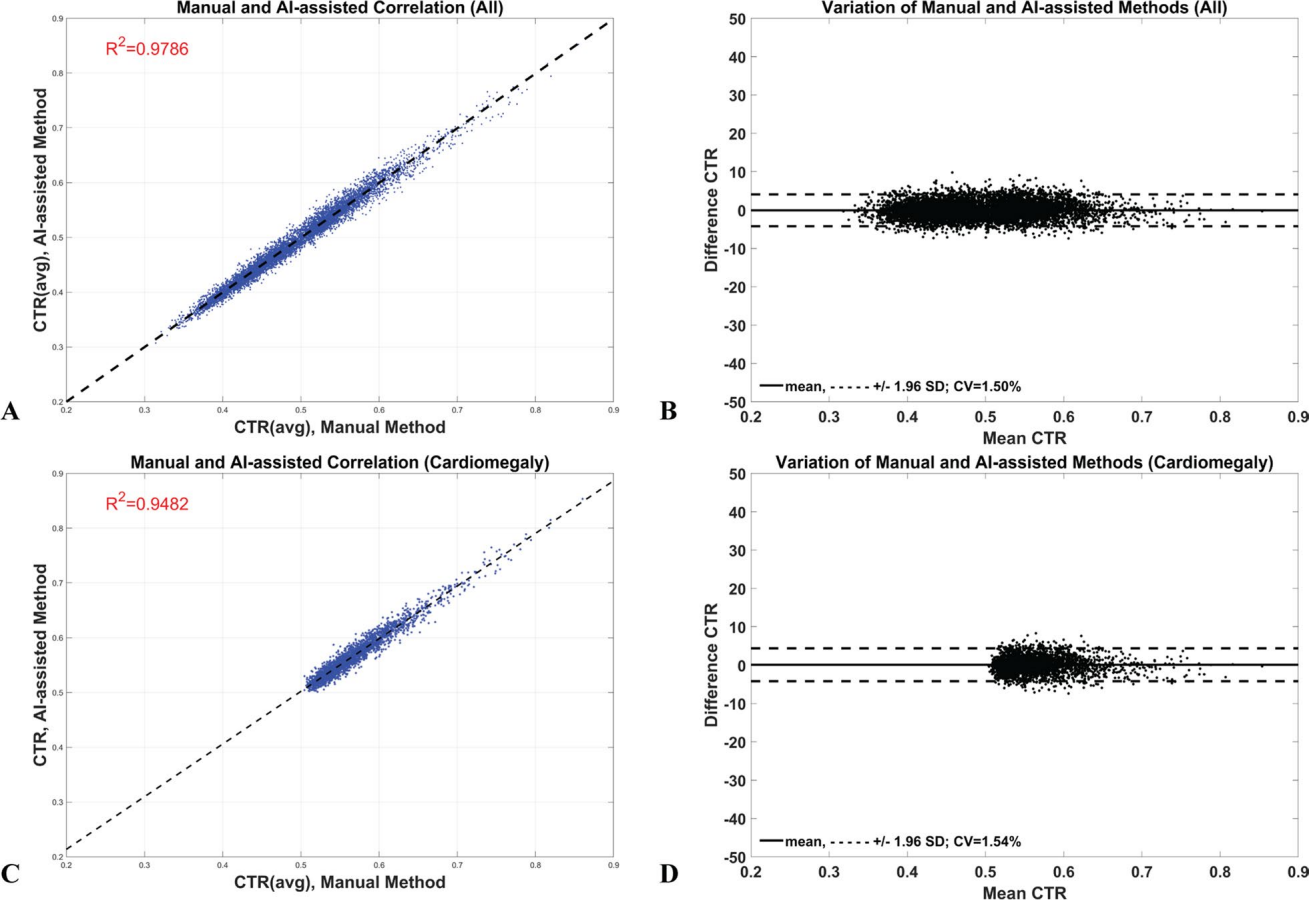
Linear correlation (**a**, **c**) and Bland–Altman (**b**, **d**) plots of Manual and AI-assisted method on all (the first row) and cardiomegaly (the last row) data. Note: These comparisons had low variation on both types of data and unlike from Manual and AI-only method (Fig. 5), their R-squared were consistency at good category (0.978 and 0.9482)

In contrast, the CVs of comparison between manual and AI-only methods were about three times higher than the inter-observer variation in the manual method on all data (5.78%), and in the cardiomegaly (5.61%) groups (Fig. 5b, d, and Table 3). Interestingly, although these two groups had similarly high variation, their coefficients of determination were noticeably different: all data was good (R^2^ = 0.79) while the cardiomegaly group was poor (R^2^ = 0.34) (Fig. 5a, c). The CVs of comparison between the manual and AI-assisted methods, on the other hand, were significantly lower than the inter-observer variation of manual method, 1.50% and 1.54% *vs* 2.13%, on all and cardiomegaly data groups, respectively (Fig. 6b, d). Furthermore, unlike the AI-only method, their coefficients of determination were in a similar excellent category (R^2^ > 0.9) (Fig. 6a, c). The AI-assisted method, therefore, had a similar variation as the manual method but the AI-only method was about three times higher than the manual one. Furthermore, these results demonstrate that the R-squared measurement is not a reliable indicator to verify the AI method.

**Table 3:** Comparison of Bias, 95% CI, and coefficient of variation (CV) of CTR measurements

Comparison	Normal and cardiomegaly data		Cardiomegaly data	
	Bias (95% CI) (%)	CV (%)	Bias (95% CI) (%)	CV (%)
Manual *vs* AI-only	−0.93 (−15.0 13.14)	5.78	−0.09 (−13.30 13.12)	5.61
Manual *vs* AI-assisted	−0.08 (−4.22 4.07)	1.50	0.08 (−4.20 4.37)	1.54

The performances of cardiomegaly classification tests are presented in Table 4. All performance metrics from manual and AI-assisted methods were comparable on all cutoff points, and all were in the excellent level (*e.g.*, AUC > 0.9 with all cutoff points around 0.5). The AI-only method gave similar outcomes only on the standard and optimum cutoffs, but provided a poor outcome on the cutoff point at the maximum sensitivity, (*e.g.*, accuracy of 34.8% with cutoff point around 0.2). The classification metrics were not reliable parameters to evaluate the performance of the AI-only method. For example, the AI-only method had almost three times higher observer and method variations as compared to the manual method, but if standard or optimum cutoffs were used, they would misleadingly suggest that the AI-only method also had the same excellent classification performance as the manual method. In contrast, if the cutoff for ruling out cardiomegaly (*i.e.*, cutoff at the maximum-sensitivity) was used then classification performance would be very poor. Thus, the cutoff criteria will dictate the outcome of the AI study in CTR measurement.

**Table 4:** Classification test on Manual, AI-only, and AI-assisted methods using cutoff points at the Standard (0.5), Optimum (maximum sensitivity and specificity), and maximum-sensitivity (Max-Sens)

Method	Test	CTR Cutoff	Sensitivity (%)	Specificity (%)	Accuracy (%)	F1	AUC
Manual	Standard	0.5	100	83.6	89.3	0.866	0.978
	Optimum	0.518	95.8	91.8	93.2	0.907	
	Max-Sens	0.505	100	86.0	90.9	0.884	
AI-only	Standard	0.5	97.5	82.8	87.9	0.849	0.962
	Optimum	0.521	90.3	91.0	90.8	0.872	
	Max-Sens	0.219	100	0.10	34.8	0.516	
AI-assisted	Standard	0.5	100	84.3	89.8	0.873	0.977
	Optimum	0.516	96.0	91.3	92.95	0.904	
	Max-Sens	0.501	100	85.0	90.2	0.876	

### Measurement time

Average CTR measurement time for the manual method was about 10.6 ± 1.5 secs per case while it was almost five times faster (2.2 ± 2.4 secs) in the AI-assisted method on all data and on the cardiomegaly group alone (data not shown). From the AI-assisted method, the measurement times were 0 s, 3.1 ± 1.8 secs, and 10.2 ± 1.3 for the excellent, good, and poor categories, respectively. The AI-assisted method then is almost five times faster to perform than the manual method. Furthermore, if the data were selected from the AI-only and manual methods using the AI’s excellent outcome criteria, then the CTR differences from the two methods will be in the range of ± 1.8% (calculated from 95% confidence interval of data). In other words, if the outcome from the AI-only method differs from the manual method by less than ± 1.8%, then its outcome can be accepted without any further interaction from the user (*i.e.*, an excellent category).

In summary, the method variations from the AI-only method were about three times higher than from the manual method. CTR calculations from the AI-only method, however, are a very useful tool to assist the user, providing a better agreement and are almost five times faster to perform. Furthermore, CV is a better parameter than R-squared or the classification performance test for the validation of AI in CTR measurement.

## Discussion

CTR derived from CXR is a valuable index for the evaluation of heart diseases, especially cardiomegaly [1–4]. To measure it, however, still requires manual operations that are user dependent and time consuming. Despite its utility, the measurement process is a burden in clinical practice. Recently, the AI method successfully provided automatic calculations of such an index and has been validated technically in various studies [9–12]. To use AI in the clinical setting, there is a need for clinical evaluation to assess the measurement agreement with the manual method. However, there have been only two published pilot studies [9, 11] with small datasets that addressed this issue.

To our knowledge, this study was the first report of observer and method variations to validate CTR measurement using AI on a large dataset (n = 7,517). Using a modified U-Net deep-learning model (*i.e.*, 2D VGG-16 U-Net) for CTR calculation, AI-only was found not to be suitable for use as an automated method of CTR measurement due to its high variations compared to the manual method. Its CTR calculations, on the other hand, can assist the user to obtain better results. Furthermore, the coefficient of determination (R^2^) or classification performance test (*e.g.*, AUC) should not be employed because it may lead the investigator to falsely conclude that the AI-only method can be employed on an automated basis. Bland–Altman plot with Covariant of Variation (CV) parameters evaluated on a large data should be utilized instead to indicate agreement between these methods.

We found that the AI-only method can provide excellent outcomes in about 40% of the data, which is a desirable result for an automated method. However, about 56% of outcomes required adjustment by the user (*i.e.* good outcome), a condition that must be improved before AI-only can be used automatically. Specifically, the AI-only method needs to be improved on heart diameter calculation which is difficult to perform because its pixel value is low, and its edges are fused with the lung borders or the thoracic spine [19]. In addition, the AI-only method also had about a 4% failure rate (*i.e.*, poor outcome) most of which was in the normal data group (97%: 290/299). In routine clinical usage, where CTR is measured only on suspected cardiomegaly cases, this failure is infrequent (9 failures in 2,517 cardiomegaly data). Nevertheless, most of the segmentation failure was on hearts with quite short diameters (*e.g.*, Fig. 3j). This may be due to an inadequate presentation of such heart data shape in the training dataset. Fine tuning the model using a local heart shape dataset should further reduce such failures.

We found that the AI-assisted method had lower inter-observer bias and variation than the manual method (CV and bias: 1.72% *vs* 2.13% and − 0.61 *vs* − 1.62). This may be due to the AI’s excellent outcome in about 40% of data which can help to improve measurement agreement. Furthermore, it is almost five-fold faster to perform than using the manual method, and increases F1 from 0.866 to 0.872 at the standard CTR cutoff point of 0.5. This clearly demonstrates the usefulness of the AI method to assist with CTR measurement. Our AI-assisted time performance was also in agreement with a recent study by Bercean et al. [9] which found a similar magnitude of time reduction (22.5 *vs* 5.1 secs, or 4.4 times). Even on a small dataset (n = 200), that study also found that the model-assisted method can improve the individual radiologist’s cardiomegaly F1 score (0.845 to 0.851) compared to the manual method.

We concluded that the classification performance test of the AI-only method was not better than from the manual method, a finding at odds with a report by Li et al. [11] that found that the sensitivity and negative-predictive values of the AI-only method were significantly better than the manual method. This may be due to two factors. First, the performance of deep learning algorithms in automated CTR measurement tasks depends on their ability to correctly locate heart and lung boundaries. In Li et al. [11], algorithms may have achieved more precise anatomical segmentations, although the authors did not provide precision metrics on an open dataset for comparison with the model we used [10]. Second, the algorithm in Li et al. [11] was trained and tested on the same dataset, while the model used in this paper was trained on an open dataset, and tested in an out-of-sample fashion. It would be useful to validate their finding by performing the classification test using their model on our dataset.

CTR measured from manual and AI-assisted methods were in substantial agreement with the reference method (CVs of 2.0 and 2.2%, respectively). The AI-only method, in contrast, had almost three times higher CVs on all comparisons. This strongly suggests that the AI-only method is not yet suitable to be employed as an automated method. However, its R^2^ of all data (normal and cardiomegaly groups) and classification performance test at the standard or optimum cutoffs were similar to other methods. This is because R^2^ measures linear association rather than agreement of data [20, 21] and measurements with highly correlated data may have poor agreement [21], as in our case. Furthermore, the correlation typically depends on the range of measure. This is why the R^2^ of the manual and AI-only methods was good in normal and cardiomegaly groups (R^2^ = 0.79; CTR data range = 0.35–0.85), but poorly correlated in the cardiomegaly group alone (R^2^ = 0.34; CTR data range = 0.52–0.85) (Fig. 5a, c). On the other hand, if the Bland–Altman plot and CV present with good agreement (Fig. 6b, d), then they are likely to be highly correlated [21] as shown in Fig. 6a, c. Thus, the agreement measurement should be employed to evaluate the compatibility of the AI to the manual method in CTR measurement study.

Classification performance tests may also be misleading because they only provide information on the performance of normal and cardiomegaly groups, and not how the methods agree. For example, Fig. 3d, f present cases where the AI-only method gave a false positive and negative result, respectively. These two data have an effect on the classification test, but most of the AI data did not have this effect (*i.e.*, AI’s CTR data did not change the classification) as shown in Fig. 3e and Table 4. Still, we obtained excellent classification performance at the standard CTR cutoff (*e.g.*, AUC = 0.902). However, if the AI-only method were employed to rule out cardiomegaly patients (*i.e.*, using CTR cutoff at the maximum sensitivity), then the method would perform poorly (*e.g.*, accuracy of 34.8%) and should not replace the manual approach. Test agreement is necessary for evaluation of the AI-only method if it is to be implemented as an automated method, and its agreement should be comparable to the manual method (CV = 2.1%).

We performed observer and method variation tests on a large dataset using only a modified U-Net Deep-Learning model because we wished to obtain baseline AI performance data. Our results, especially the manual measurement of 7,517 CXRs, will serve as a reference to evaluate other state-of-the-art AI models [22]. Our plan is to test these models on our dataset and accept the AI outcome only if it differs from our manual results by less than ± 1.8% (*i.e.*, an excellent category where the user can accept its outcome without adjustment). Any model with > 70% acceptance rate will be studied prospectively in a clinical setting and evaluated by our radiologists. Furthermore, at such an acceptance rate, we will perform another retrospective study in our PACS data (around one million CXR images). Such a pioneering study would provide more insight into CTR values and useful information for clinicians.

There were some limitations in our dataset and methods. We used only normal and cardiomegaly data and there was no data from other pathologies, such as the fat pad of the pericardium or pleural effusion. These pathologic conditions may limit the DL model’s ability to segment heart and lung, and may lower the performance of CTR measurement. Such data should be included in future studies to better evaluate the performance of the model. Furthermore, we only investigated adult cases, but evaluation of CTR measurement by AI in pediatric cases is needed. Next, we used only a publicly available dataset. Future studies using local datasets are needed to improve the model’s performance. Finally, unlike most deep learning for CXR analysis studies, this study did not address the question of how AI can be trained to match human performance in CTR measurement, but focused on assessing the extent to which deep learning methods can benefit the radiologists’ practice in a clinical setting. Future studies may focus more on the patterns of errors generated by the algorithms and suggest ways to improve their accuracy.

## Conclusion

We conclude that AI should be employed to assist radiologists to perform CTR measurement because it can significantly reduce variations and is almost five-fold faster than the manual method. However, AI alone is not yet suitable for automated measurement due to its high variations. Agreement of measurement, like the Bland–Altman plot and CV should be used to evaluate the comparability of AI to the manual method, while the coefficient of determination or classification performance test should be used with caution because it is not a reliable indicator.

## Data Availability

The datasets generated during and/or analyzed during the current study are not publicly available due to patients’ data privacy policy by our hospital but are available from the corresponding author on reasonable request.

## Abbreviations

AI: Artificial Intelligence
DL: Deep learning
CTR: Cardiothoracic ratio
CV: Coefficient of variation
CXR: Chest radiography
PACS: Picture archiving communication system
R-squared: Coefficient of determination
